# Effects of editing *DFR* genes on flowers, leaves, and roots of tobacco

**DOI:** 10.1186/s12870-023-04307-7

**Published:** 2023-07-05

**Authors:** Jiarui Jiang, Haitao Huang, Qian Gao, Yong Li, Haiying Xiang, Wanli Zeng, Li Xu, Xin Liu, Jing Li, Qili Mi, Lele Deng, Wenwu Yang, Jianduo Zhang, Guangyu Yang, Xuemei Li

**Affiliations:** 1 Technology Center, China Tobacco Yunnan Industrial Co. LTD, No. 181 Hongjin Road, Kunming, 650000 Yunnan Province China; 2 Jiangsu Academy of Agricultural Sciences, No. 50 Zhongling Street, Nanjing, 210014 Jiangsu Province China

**Keywords:** *DFR*, Gene editing, CRISPR, Cas9, Flavonoid metabolism

## Abstract

**Background:**

*DFR* is a crucial structural gene in plant flavonoid and polyphenol metabolism, and *DFR* knockout (*DFR*-KO) plants may have increased biomass accumulation. It is uncertain whether *DFR*-KO has comparable effects in tobacco and what the molecular mechanism is. We employed the CRISPR/Cas9 method to generate a knockout homozygous construct and collected samples from various developmental phases for transcriptome and metabolome detection and analysis.

**Results:**

*DFR*-KO turned tobacco blossoms white on homozygous tobacco (Nicotiana tabacum) plants with both *NtDFR1* and *NtDFR2* knockout. RNA-seq investigation of anthesis leaf (LF), anthesis flower (FF), mature leaf (LM), and mature root (RM) variations in wild-type (CK) and *DFR*-KO lines revealed 2898, 276, 311, and 101 differentially expressed genes (DEGs), respectively. *DFR*-KO primarily affected leaves during anthesis. According to KEGG and GSEA studies, *DFR*-KO lines upregulated photosynthetic pathway carbon fixation and downregulated photosystem I and II genes. *DFR*-KO may diminish tobacco anthesis leaf photosynthetic light reaction but boost dark reaction carbon fixation. *DFR*-KO lowered the expression of pathway-related genes in LF, such as oxidative phosphorylation and proteasome, while boosting those in the plant–pathogen interaction and MAPK signaling pathways, indicating that it may increase biological stress resistance. *DFR*-KO greatly boosted the expression of other structural genes involved in phenylpropanoid production in FF, which may account for metabolite accumulation. The metabolome showed that LF overexpressed 8 flavonoid metabolites and FF downregulated 24 flavone metabolites. In *DFR*-KO LF, proteasome-related genes downregulated 16 amino acid metabolites and reduced free amino acids. Furthermore, the DEG analysis on LM revealed that the impact of *DFR*-KO on tobacco growth may progressively diminish with time.

**Conclusion:**

The broad impact of *DFR*-KO on different phases and organs of tobacco development was thoroughly and methodically investigated in this research. *DFR*-KO decreased catabolism and photosynthetic light reactions in leaves during the flowering stage while increasing carbon fixation and disease resistance pathways. However, the impact of *DFR*-KO on tobacco growth steadily declined as it grew and matured, and transcriptional and metabolic modifications were consistent. This work offers a fresh insight and theoretical foundation for tobacco breeding and the development of gene-edited strains.

**Supplementary Information:**

The online version contains supplementary material available at 10.1186/s12870-023-04307-7.

## Introduction

Dihydroflavonol 4-reductases (*DFR*s), controlled by various transcription factors, are important flavonoid biosynthetic enzymes that contribute to the biosynthesis of flavonoids, anthocyanins, and proanthocyanidins and are involved in the catalytic reaction of anthocyanins from dihydroflavonol [1–4]. Different flower colors in different plant species depend on the specific selectivity of *DFR*s for three dihydroflavonols (DHK, DHQ and DHM) [5, 6]. The catalytic property of *DFR*s has been found to be a limiting factor in floral color variation [7–9]. Overexpression of *DFR* in tobacco elevates levels of polyphenols and enhances stress resistance; for example, the free radical scavenging activity was improved in transgenic tobacco, and transgenic lines showed resistance against drought stress, oxidative stress, abscisic acid and infestation by the tobacco leaf cutworm *Spodoptera litura* [10, 11]. In addition, studies have found that *DFR* also acts as a flavanone 4-reductase (flavanone 4-reductase, FNR), catalyzing the production of flavin-4-alcohol (flavon-4-ols). Forming 3-deoxyanthocyanins (3-deoxyantho-cyanidin) under the action of other enzymes makes the plants orange–red; moreover, 3-deoxyanthocyanins and their intermediate metabolite pentahydroxyflavonoids (luteoforol) can also be used as defensive substances against fungi and bacteria [12, 13]. *DFR* is also associated with stress resistance; therefore, tobacco (*Nicotiana tabacum*) with edited *NtDFR* anthocyanin genes was investigated for its effects on different organs and use in agriculture.

Tobacco (*Nicotiana tabacum*), a natural allotetraploid, was derived from hybridization between *N. sylvestris* and *N. tomentosiformis* [14]. Studies of the *DFR* gene in tobacco mainly focus on anthocyanin metabolism and flower color change, and there is almost no research on organs in other parts of tobacco. As an important cash crop and model organism, to study the effects of *DFR* gene knockout on tobacco flavonoid metabolism and biological stress resistance, we obtained knockout homozygous constructs by using the CRISPR/Cas9 system construct and collected samples from different sites at different developmental periods for transcriptome and metabolome detection and analysis.

## Materials and methods

### Plant materials

**Fig. 1:**
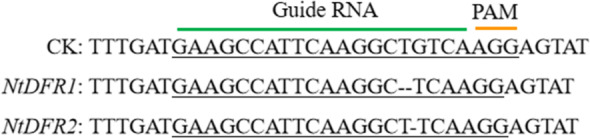
CRISPR/Cas9-mediated *DFR* gene mutation in *Nicotiana tobacum*

Tobacco (*Nicotiana tabacum* cv HongHuaDaJinYuan) mutants of *NtDFR1*/*NtDFR2* were obtained by gene editing mediated by the CRISPR/Cas9 system. We replaced the gRNA with "GAAGCCATTCAAGGCTGTCAAGG" and obtained the mutant, referring to the study by Gao et al. [15]. According to the genomic analysis results, the mutant contained a frameshift mutation in the *DFR* gene, and the mutation form contained either deletion of G (Fig. 1).

The mutant (*DFR*-KO) and wild-type (CK, donated to Yunnan Tobacco Agricultural Research Institute) (60) plants were grown under controlled conditions in Shilin, Yunnan, China (N24°46′27.55″, E103°17′18.83″; height: 1688.58 m; average annual temperature: 13 ~ 24 °C). To eliminate the influence of individual differences, the sampling used the mixed sample method, and the flower, leaf and root samples of three tobacco plants were mixed into one sample individually. All samples were frozen in liquid nitrogen immediately when removed from the plant and stored at − 80 °C until further use. The sample information is shown in Table 1.

**Table 1:** The sample information

	**Analysis part**	**Growth period**	**Code name**	**Sample numbers**
Wild-type tobacco (CK)	Flower	Flowering period	CK-FF	3
	Leaf	Flowering period	CK-LF	3
		Maturation period	CK-LM	6
	Root	Flowering period	CK-RF	3
		Maturation period	CK-RM	3
Gene knockout mutants (*DFR*-KO)	Flower	Flowering period	*DFR*-FF	6
	Leaf	Flowering period	*DFR*-LF	6
		Maturation period	*DFR*-LM	6
	Root	Flowering period	*DFR*-RF	3
		Maturation period	*DFR*-RM	3

### Tobacco transformation and acquisition of homozygotes

The constructed vectors were introduced into *Agrobacterium tumefaciens* (LBA4404) by the freeze–thaw method for transformation of tobacco. Leaf discs from 6-week-old tobacco plants were infected with *A. tumefaciens* harboring the vectors and were then plated onto regeneration medium (4.4 g MS medium, 30 g sucrose, 8 g agar, 2 mg/L 6-benzylaminopurine, and 0.5 mg/L 1-naphthaleneacetic acid, pH 5.6) containing 250 mg/L sodium carbenicillin and 50 mg/L kanamycin sulfate [16]. Drug-resistant seedlings were obtained and screened by molecular detection, and the T1/T2 generation of self-homozygous seedlings was obtained. Homozygotes for target editing with no transgenic labels were finally obtained.

### Metabolomics analysis

#### Metabolite extraction

The plant samples for metabolomics analysis were extracted with a mixture of MeOH and H_2_O (v/v = 80:20). Metabolites were extracted from 100 mg sample with 1.0 mL extractant by vortexing for 20 min [17]. The samples were then centrifuged at 3802 g for 10 min. The extracts were filtered through a 0.22-μm hydrophobic membrane before metabolomic analysis [18].

##### LC-QTOF/MS

The metabolic profile of plant tissue samples was acquired using LC-QTOF/MS consisting of a Shimadzu LC-20A LC system (HPLC) and a Sciex 5600 triple TOF MS/MS system (QTOF/MS) equipped with an electrospray DuoSpray ion source operating in the positive and negative ionization modes [18]. The separation was performed on a column (XSelect HSS T3 (4.6 × 150 mm, 3.5 μm)). The mobile phase consisted of 0.1% formic acid/water (A) and MeCN (B) for the positive ionization mode and water with 5 mM ammonium acetate (A) and MeCN (B) for the negative ionization mode. Under both modes, the gradient mobile phase was programmed as follows: 0–3 min, 10% B; 3–21 min, 10%-95% B; 21–28 min, 95% B; 28–28.1 min, 10% B; 28.1–34 min, 10% B [18]. The QTOF/MS full-scan mode, combined with information-dependent analysis (IDA), was selected, and the mass scan range was set as *m/z* 50–1000. The source voltages for the positive and negative ionization modes were 5500 V and 4500 V, respectively. The flow pressures of the curtain gas, nebulizer (GAS1), and heater gas (GAS2) were 25, 50 and 50 psi, respectively [18]. The collision energy (CE) was set as 30 V and -30 V for the positive and negative modes, respectively [18]. Automatic *m/z* calibration was performed by using an automated calibration delivery system (CDS) in every 5-sample interval for the positive and negative ionization modes.

#### Metabolomics bioinformatics analysis

The raw data acquired by LC-QTOF-MS were imported into MS-DIAL software for peak collection, baseline filtering and baseline calibration, peak alignment, deconvolution analysis and peak identification [19]. The aligned peak table containing the information on *m/z*, retention time and peak area was acquired. Compound identification was achieved through analyses of the retention time, mass accuracy, and isotope ratio along with MS/MS similarity matching to libraries from publicly available databases, including MassBank, LipidBlast, GNPS and MetaboBASE [19, 20]. Additionally, some compounds with commercial standards were used for identification. The matrix of *m/z* areas was analyzed based on two multivariate data analysis methods, principal component analysis (PCA) and partial least-squares discriminant analysis (PLS-DA), which were performed by using MATLAB software. The data matrix was normalized to a unit vector and mean-centered before the PCA and PLS-DA analyses. The significant difference in plant metabolites was selected based on the principles of the variable importance in projection (VIP) value larger than 1 and t test at the 95% level. MS-FINDER software was used to identify the metabolites of IMI for structure elucidation using MS and MS/MS spectra of unknown peaks [21]. The Kyoto Encyclopedia of Genes and Genomes (KEGG) database was used to study metabolomics pathways of biomarkers with a significant difference by using the MetaboAnalyst tool (http://www.metaboanalyst.ca/) [22]. The hypergeometric test was selected for overrepresentation analysis, and the algorithm of relative betweenness centrality was used for pathway topology analysis in pathway analysis [23].

### Transcriptomics analysis

#### RNA extraction and sequencing

The total RNA from each sample was extracted with a FastPure Plant Total RNA Isolation Kit (Vazyme, Nanjing, China), and RNA integrity was confirmed by 1.5% agarose gel electrophoresis. BGI Genomics Co., Ltd. (Wuhan, China) conducted all library preparation and sequencing.

#### Transcriptomics bioinformatics analysis

The raw data contained reads of low quality, reads with adaptor sequences and reads with high levels of N bases. Those reads were filtered by the DNBSEQ Sequencing Platform before data analysis to ensure the reliability of the results. We used HISAT to align the clean reads to the reference genome from NCBI (*Nicotiana tabacum*, reference genome version: GCF_000715135.1_Ntab-TN90). We used Bowtie2 to align the clean reads to the reference genes. Differentially expressed genes (DEGs) were identified with DESeq2 to make comparisons between groups. Genes were considered differentially expressed if they met the criteria |log2FC|> = 1 and Q value <  = 0.05. R was used to perform correlation analyses of these DEGs. The GSEA method used in this study was the GSEA official R package with default parameters.

According to the KEGG pathway annotation classification, the phyper function in R software was used to perform the enrichment analysis and calculate the P value, and the Q value was obtained by PDR correction of the P value. Generally, a Q value <  = 0.05 indicated significant enrichment.

#### Expression analysis by quantitative real-time PCR (qRT–PCR)

Primers were designed using Primer Express 3.0 software and full-length cDNA of candidate genes. The primer length was 18–22 bp, the amplified fragment length was 80–150 bp, and the Tm value was 55–65 °C. The primers with the highest score in the Primer Express 3.0 list were considered suitable quantitative primers as long as the BLAST result of one upstream and downstream primer was only the candidate gene (to ensure the specificity of primers) after BLAST comparison in the tobacco genome database. The commonly used internal reference genes L25, 26S and ACTIn were compared, all of which were relatively uniform without significant differences [24]. L25 was selected as the internal reference gene in this experiment (Table 2).qRT–PCR was conducted with a LightCycler 480 II (Roche, Germany). The primers for qPCR were designed with L25 as an internal reference gene. The qPCR system (20 μL) was as follows: 2 × SYBR Green Master 10 μL, 10 μmol/L forward and reverse primers 0.5 μL, cDNA template 2 μL, and water to 20 μL. The qPCR conditions were as follows: 95 °C for 10 min; 95 °C for 10 s, 62 °C for 15 s, and 72 °C for 20 s for a total of 45 cycles; 95 °C for 5 s and 65 °C for 1 min; and heating to 97 °C at 0.1 °C/s. After the reaction, according to the *Ct* value obtained, the relative expression level of the gene was calculated by the 2^−∆∆Ct^ method, and the fold change in the target gene was calculated by the 2^−∆∆Ct^ method.

**Table 2:** Primers for target gene expression levels

ID	qRT–PCR primers
*NtDFR1*	F: CCCGTGGTGTATTTTTCGCC
	R: GACAAGTCTCAATGGCCCCT
*NtDFR2*	F: GATACTGGCAGAGAAGGCCG
	R: GGAATGTAGGCGCGAGGAAT
L25	F: CCCCTCACCACAGAGTCTGC
	R: AAGGGTGTTGTTGTCCTCAATCTT

#### Anthocyanin content determination by HPLC

Freeze-dried samples of approximately 1.0 g per sample were determined according to the Ministry of Agriculture standard “High-performance liquid chromatography for measuring anthocyanins in plant-derived foods (NY/T 2640–2014)”.

## Results

### Effects of *DFR* knockout on the phenotype and expression validation of the* DFR*

**Fig. 2:**
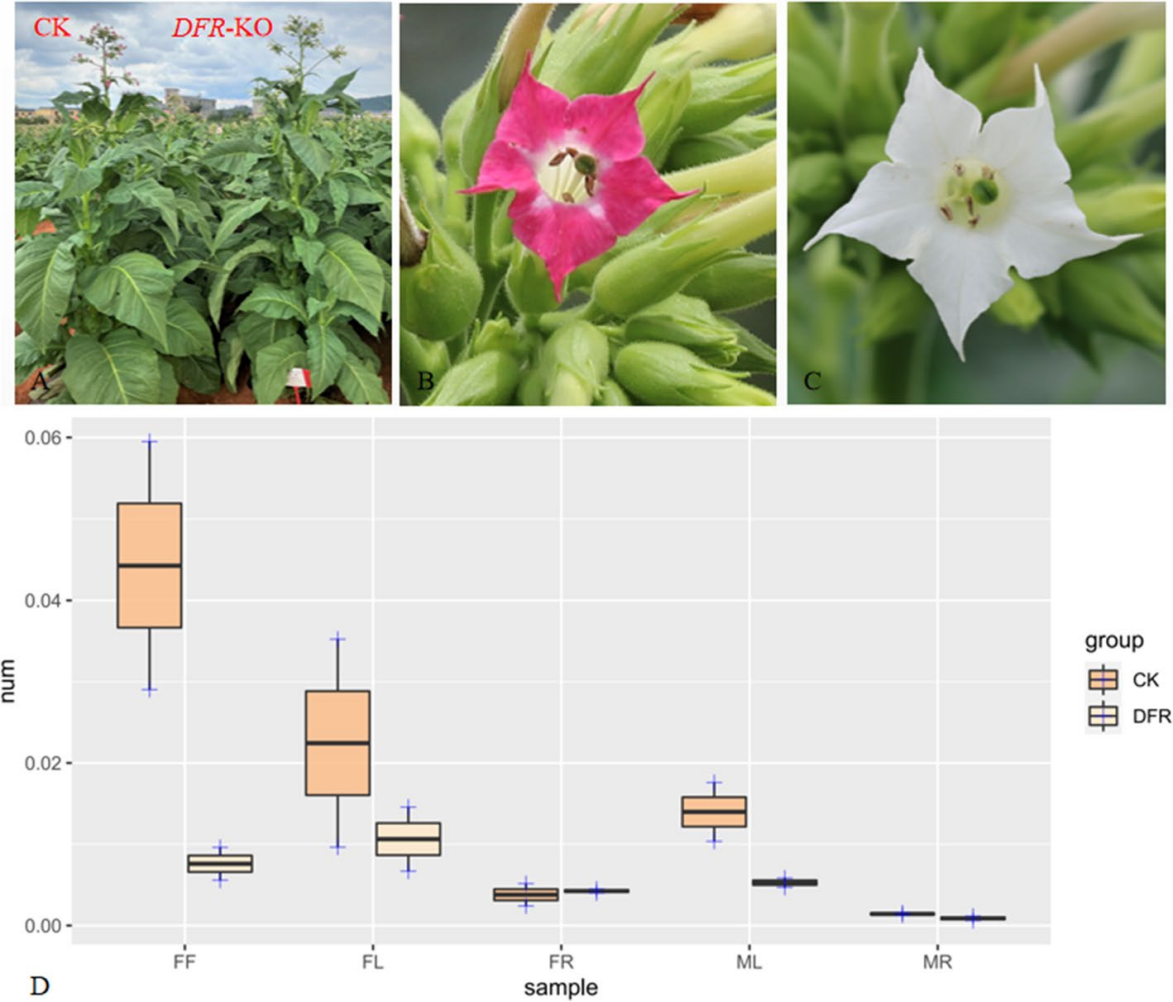
Phenotypic and *DFR* gene qPCR validation of CK and *DFR*-KO. **A** Comparison of CK and *DFR*-KO growth. **B** Corolla of CK. **C** Corolla of *DFR*-KO. D: qPCR validation of the *DFR* gene in different analysis parts and growth periods of CK and *DFR*-KO. Sample names: FF (flower of flowering period), LF (leaf of flowering period), RF (root of flowering period), LM (leaf of maturation period) and MR (root of maturation period)

The *DFR*1/*DFR*2 gene in tobacco was knocked out in this research utilizing the CRISPR/cas9 platform, and the largest difference between before and after knockdown samples was the color of the tobacco flower. The *DFR* knockout (*DFR*-KO) has white flowers, while the wild type (CK) has red blooms (Fig. 2B-C). Meanwhile, there was no growth difference between CK and *DFR*-KO (Fig. 2A). qPCR was used to confirm *DFR* expression in multiple organs of the *DFR*-KO line, and *DFR* was dramatically downregulated in flower (FF), leaf (LF), and leaf (LM) during anthesis (Fig. 2D).

### Overview of transcriptome analysis for *DFR* knockout mutants

**Fig. 3:**
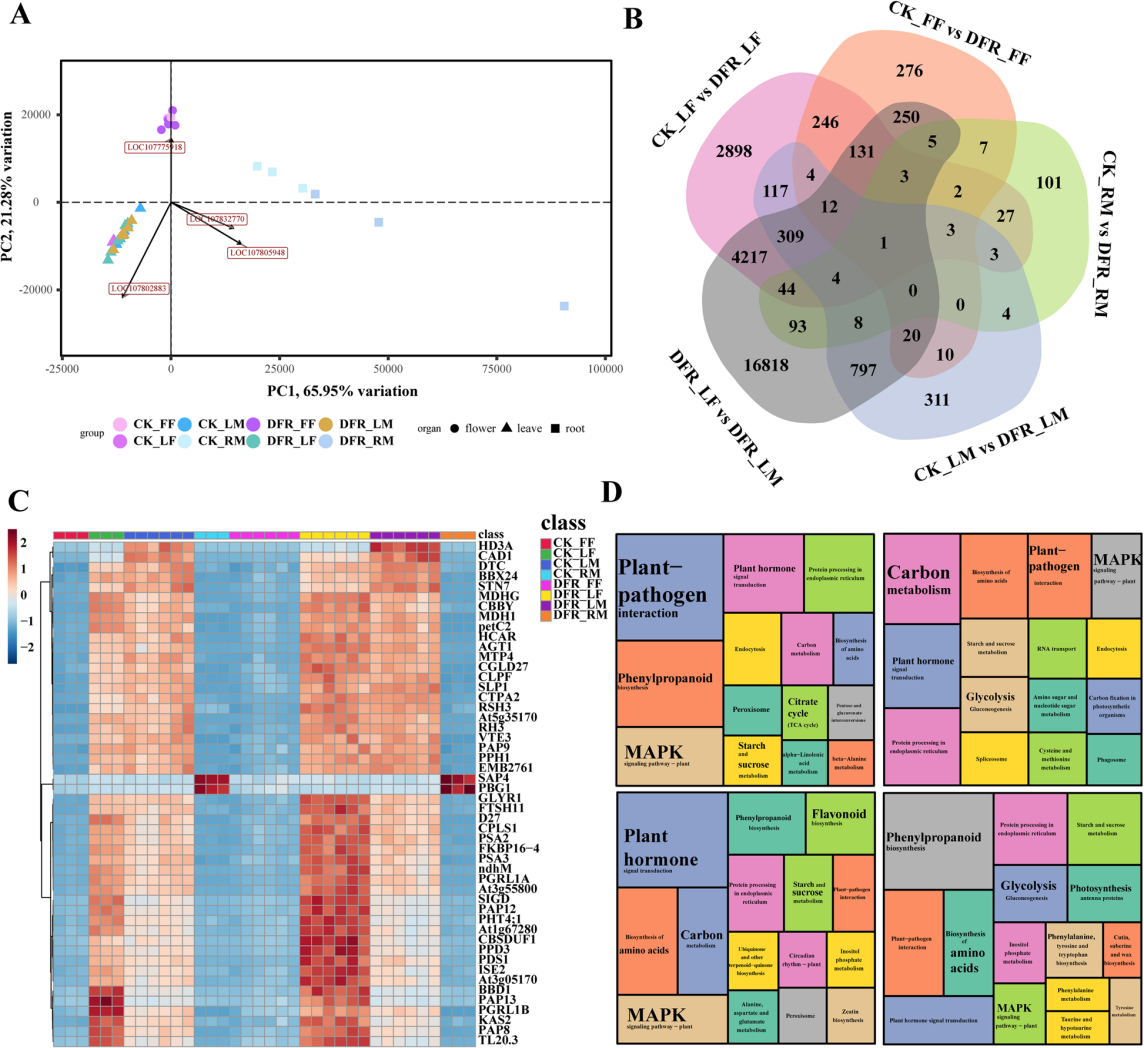
Transcriptome data analysis statistics. **A** PCA of transcriptome data consistency across all samples. PC1 indicates the first principal component, and PC2 indicates the second principal component. **B** Venn diagram of differentially expressed genes. **C** Gene clustering heatmap. **D** KEGG enrichment analysis of DEGs in the comparisons of CK_FF vs. *DFR*_FF, CK_LF vs. *DFR*_LF, CK_LM vs. *DFR*_LM, and CK_RM vs. *DFR*_RM, where the size of the area represents the number of enriched genes. Sample names: FF (flower of flowering period), FL (leaf of flowering period), FR (root of flowering period), ML (leaf of maturation period) and MR (root of maturation period)

Flowering stage flowers (FF), flowering stage leaves (LF), mature leaves (LM), and mature roots (RM) were investigated for RNA-seq in CK and *DFR*-KO, respectively, to further investigate the transcriptional alteration patterns in *DFR*-KO. Principal compoent analysis (PCA) revealed that PC1 (describes the most obvious features in the multidimensional data matrix) accounted for most of the differences between leaves and flowers, while PC2 (represents the most significant features in the data matrix except PC1) accounted for the majority of the differences between leaves and flowers (Fig. 3A). The difference between the *DFR*-KO and CK was particularly noticeable in RM (Fig. 3A). Further differential analysis identified 276 distinct DEGs in the CK-FF and *DFR*-FF groups and 311 unique DEGs in the CK-LM and *DFR*-LM groups and CK-RM and *DFR*-RM groups (Fig. 3B). They had 101 different DEGs among them. There were 2898 distinct DEGs in the CK-LF and *DFR*-LF groups (Fig. 3B). DEGs in LF were shown to be mostly associated with pathways such as carbon metabolism, carbon fixation in photosynthesis, and plant–pathogen interaction, according to functional analysis (Fig. 3D). Plant–pathogen interaction and phenylpropanoid biosynthesis are the processes primarily altered by *DFR* deletion in FF (Fig. 3D). Other critical pathways were impacted in both LF and LM (Fig. 3D). The global DEG expression heatmap also demonstrated the diverse impacts of *DFR* knockdown on various organs (Fig. 3C).

### GSEA of the up- and downregulated pathways in leaf samples at the flowering stage

**Fig. 4:**
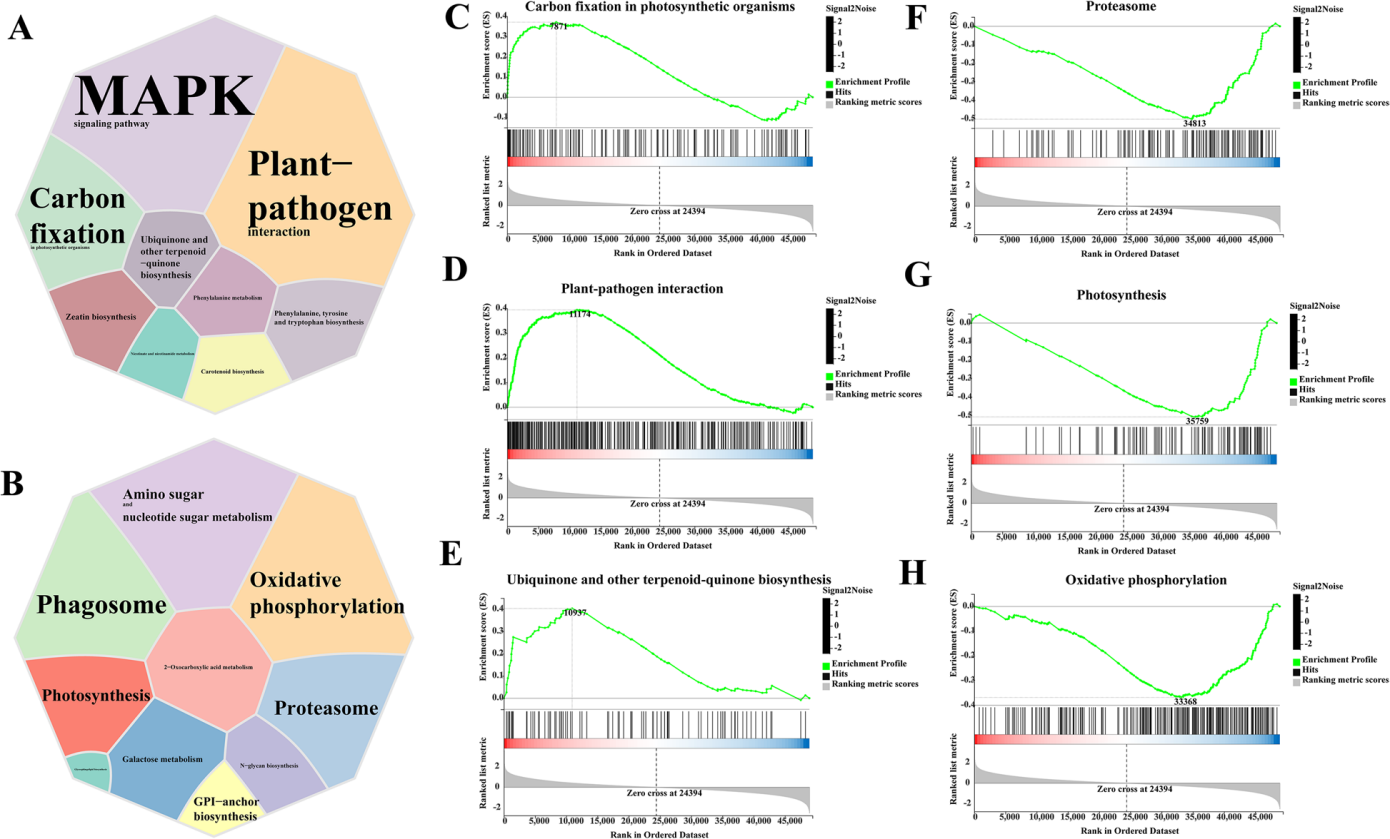
**A**-**B** GSEA of the up- and downregulated pathways in leaf samples of the flowering stage. **C**-**H** GSEA plot of carbon fixation in photosynthetic organisms, plant–pathogen interaction, plant–pathogen interaction, proteasome, photosynthesis, and oxidative phosphorylation

Previous research found that the flowering stage leaf (LF) is the most influenced region of tobacco after *DFR* knockout; thus, we used GSEA to investigate the functional changes in upregulated and downregulated genes in *DFR*-KO. The majority of the upregulated genes in *DFR*-KO were associated with the MAPK (mitogen-activated protein kinase) signaling pathway, plant–pathogen interaction, carbon fixation in photosynthetic organisms, and the manufacture of plant–pathogen interaction (Fig. 4A, C-E). The majority of downregulated genes in *DFR*-KO were associated with the proteasome, photosynthesis, and oxidative phosphorylation (Fig. 4B, F–H).

### *DFR* knockdown alters the expression profile of up- and downregulated genes in leaves during the flowering (LF) stage

**Fig. 5:**
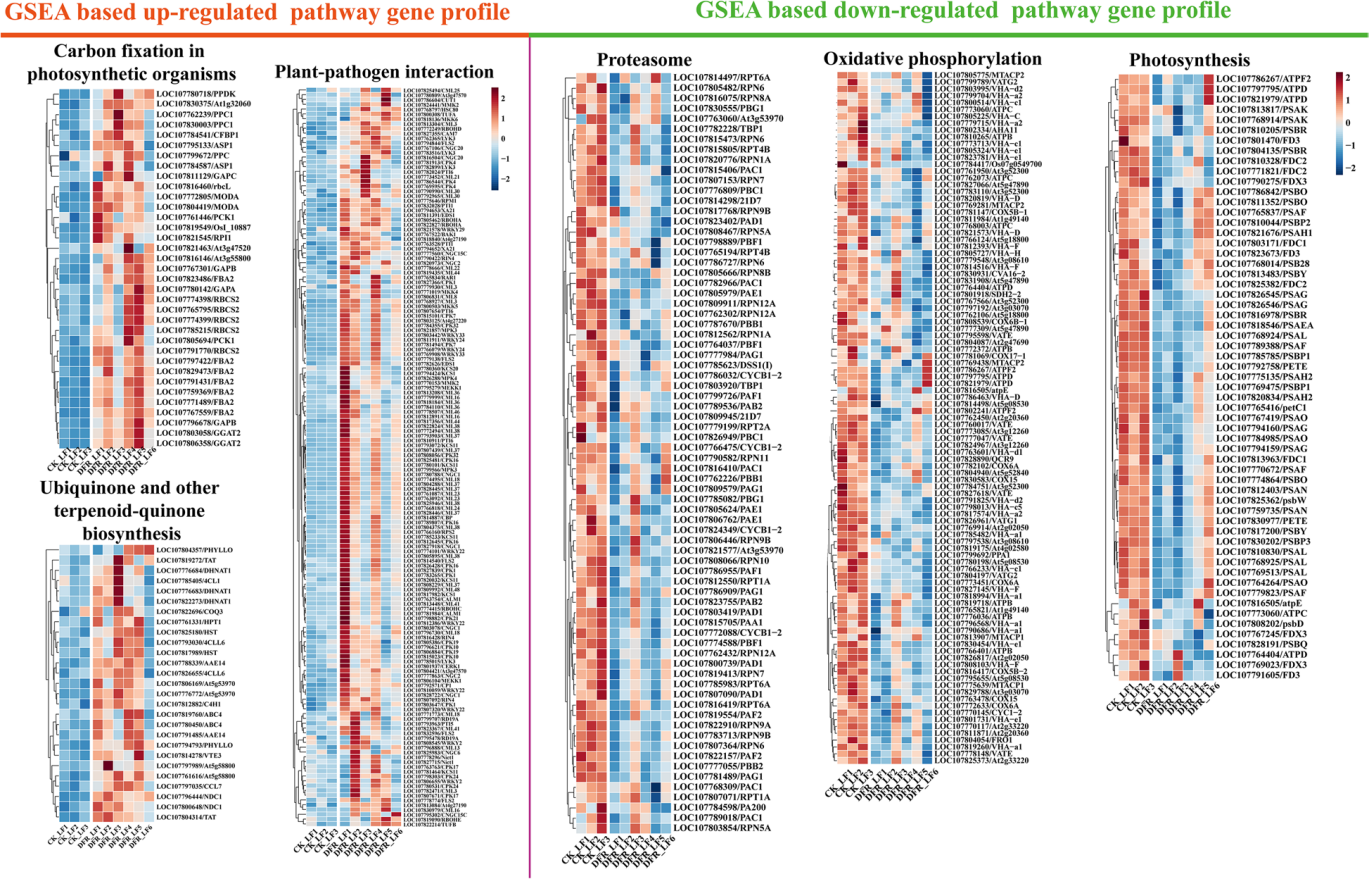
GSEA-based up- and downregulated pathway gene profiles

The essential genes in the plant–pathogen interaction, carbon fixation in photosynthetic organisms, ubiquinone and other terpenoid-quinone production, proteasome, photosynthesis, and oxidative phosphorylation pathways were investigated to further investigate the particular gene change patterns produced by *DFR*-KO (Fig. 5). Key genes involved in carbon fixation in photosynthetic organisms, such as *RBCS2*, *FBA2*, *RBCL*, *GAPA*, *GAPB*, *GGAT2*, and *PCK1*, were shown to be upregulated in *DFR*-KO. *DFR*-KO increased the expression of key genes involved in the production of ubiquinone and other terpenoid quinones, including *4CL1*, *TAT*, *HPT1*, *4CLL6*, *HST*, and others. Many critical genes involved in plant–pathogen interactions, including *FLS2*, *CML25*, *RBOHA*, *WRKY29*, *MKK5*, *WRKY33*, *WRKY24*, and others, were shown to be upregul*ated in DFR-KO. In contrast, we discovered* that photosystem I genes such as *PSAEA, PSAF, PSAG, PSAH1, PSAH2, PSAK, PSAL, PSAN*, and *PSAO* were considerably downregulated in *DFR*-KO. Photosystem II-related genes, including *PSBD*, *PSBW*, *PSB28*, *PSBO*, *PSBP1*, *PSBP2*, *PSBP3*, *PSBQ*, *PSBR*, *PSBY*, and others, were similarly considerably downregulated in *DFR*-KO. At the same time, the most important oxidative phosphorylation pathway genes *ATPB*, *ATPC*, *ATPD*, *ATPF2*, *COX15*, *COX171*, *COX5B1*, *COX5B2*, *COX6A*, *COX6B1*, *VHAC*, *VHAD*, *VHAF*, and *VHAH* were downregulated in *DFR*-KO, suggesting that *DFR*-KO may have significantly decreased aerobic respiration in tobacco leaves. Furthermore, genes associated with proteasome function, such as *RPN10*, *RPN11*, *RPN12A*, *RPN1A*, *RPN5A*, *RPN6*, *RPN7*, *RPN8A*, *RPN8B*, *RPN9A*, *RPN9B*, *RPT1A*, *RPT2A*, *RPT4B* and *RPT6A*, were downregulated in *DFR*-KO, indicating a reduced amount of protein degradation.

### Gene functional analysis and expression profile of *DFR*-KO in Mature Leaf (LM)

**Fig. 6:**
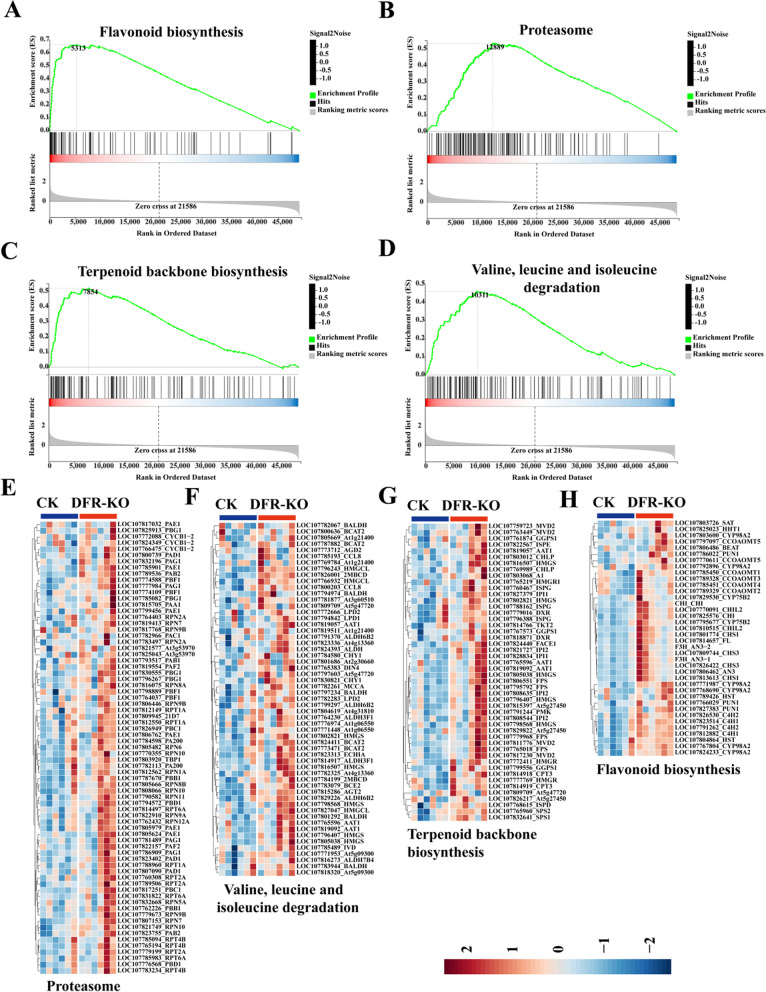
GSEA of LM transcriptomes in CK and *DFR*-KO mice. **A**-**D** GSEA plot of flavonoid biosynthesis, proteasome, terpenoid backbone biosynthesis, and valine, leucine, and isoleucine degradation. **E-H** GSEA-based proteasome, valine, leucine, and isoleucine degradation, terpenoid backbone biosynthesis, flavonoid biosynthesis pathway gene profile

GSEA was performed on LM transcriptomes in CK and *DFR*-KO to further investigate the dynamics of transcriptome alterations in mature leaves (LM) in *DFR*-KO. *DFR*-KO upregulated genes involved in flavonoid biosynthesis, proteasome, terpenoid backbone biosynthesis, and valine, leucine, and isoleucine degradation in LM (Fig. 6A-D). The modifications in the route in the LM stage were quite different from those in the LF stage. The majority of proteasome-related genes were downregulated in the LF stage but upregulated in the LM stage (Fig. 6B, E). *RPN1A*, *RPN2A*, *RPN5A*, *RPN6*, *RPN7*, *RPN8A*, *RPN8B*, *RPN9A*, *RPN9B*, *RPT1A*, *RPT2A*, *RPT4B*, *RPT6A*, and other proteasome-related genes were upregulated in *DFR*-KO, implying enhanced protein breakdown. At the same time, important flavonoid synthesis genes such as *C4H1*, *C4H2*, *CCOAOMT1*, *CCOAOMT2*, *CCOAOMT3,*, *CCOAOMT4*, *CCOAOMT5*, *CHI*, *CHIL2*, *CHS1*, *CHS3*, and others were all upregulated in *DFR*-KO (Fig. 6A, H). Similarly, critical structural genes involved in terpenoid synthesis, such as *DXR*, *FACE1*, *FPS*, *GGPS1*, *HMGR*, *HMGR1*, *HMGS*, *IPI1*, *IPI2*, *ISPD*, *ISPE*, *ISPG*, *MVD2*, *PMK*, *SPS1*, *SPS2*, and *TKT2*, were upregulated in *DFR*-knockout mice (Fig. 6C, G). Genes involved in the degradation of valine, leucine, and isoleucine, such as *BALDH*, *BCAT2*, *BCE2*, *CCL8*, *CHY1*, *DIN4*, *ECHIA*, *HMGCL*, *HMGS*, *IVD*, *LPD1*, *LPD2*, and *MCCA*, were also upregulated in *DFR*-KO (Fig. 6D, F).

### Expression changes in anthocyanin and flavonoid metabolism-related genes in flower samples after *NtDFR* knockout

**Fig. 7:**
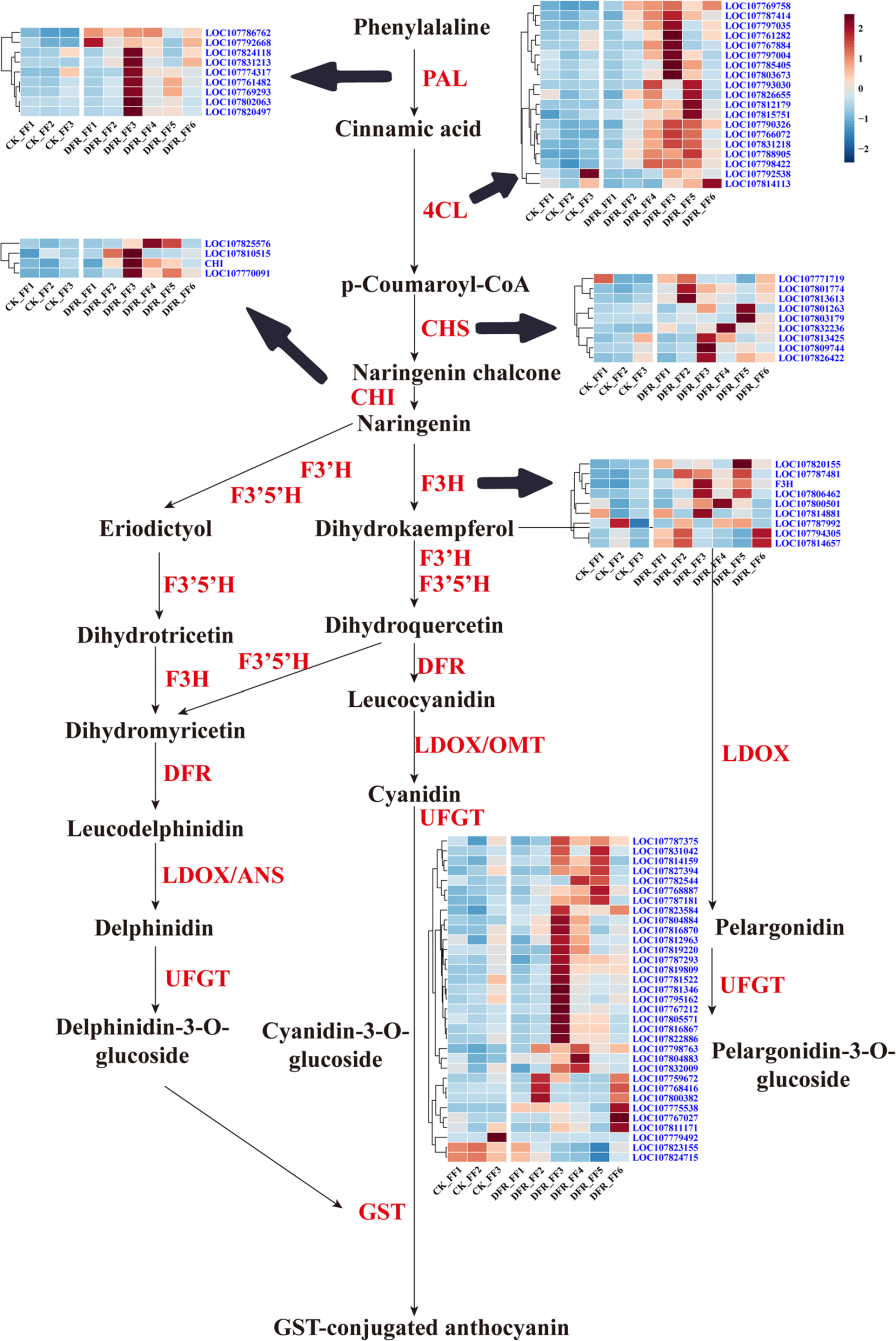
Heatmap diagrams of the relative expression levels of anthocyanin biosynthesis-related structural genes [25]. *PAL*: phenylalanine ammonia lyase; *C4H*: cinnamate 4-hydroxylase; *4CL*: 4-coumaroyl: CoA ligase; *CHS*: chalcone synthase; *CHI*: chalcone isomerase; *F3H*: flavanone 3-hydroxylase; *DFR*: dihydroflavonol-4-reductase; *OMT*: O-methyltransferase; *GST*: glutathione S-transferase; the shades of color in the heatmap represent the TPM expression after log normalization

The tobacco flower color of the *DFR*-KO strain changed from red to white, and *DFR* gene expression in the flower was also dramatically reduced (Figure S1). *DFR* is a flavonoid metabolism enzyme that can catalyze dihydroquercetin, dihydrokaempferol, eriodictyol, garbanzol (5-deoxydihydrokaempferol), and dihydrofisetin. We recreated the flavonoid and anthocyanin metabolic pathways in tobacco and investigated the impact of *DFR*-KO on additional structural genes (Fig. 7). The expression of six essential genes, *PAL*, *4CL*, *CHS*, *CHI*, *F3H*, *DFR*, and *UFGT*, was shown to be dramatically altered in *DFR*-KO. There were 9 *PAL*, 4 *CHI*, 19 *4CL*, 9 *CHS*, 9 *F3H*, and 33 *UFGT* genes found to be upregulated in the flowers of *DFR*-KO. This might suggest that *DFR* deletion causes the accumulation of its catalytic low substances and that the buildup of low substances increases the expression of other structural genes.

### Multiple tissue and multistage metabolite changes induced by *DFR* knockout

The findings suggest that *DFR*-KO causes widespread alterations in transcriptome gene expression. Nontargeted metabolome detection using LC–MS/MS was conducted on all samples matching the transcriptome to investigate the influence of these gene expression variations on metabolites in different organs of tobacco. A total of 316 metabolites were found in all samples (Table S1). The findings of the differential analysis revealed that 24 flavonoid metabolites, including cyanidin-3-O-galactoside, eriodictyol-7-O-glucoside, naringenin-7-O-glucoside, quercetin, and others, were downregulated in *DFR*-KO at the FF stage (Table S1). *DFR*-KO, on the other hand, increased the levels of 8 flavonoid metabolites while decreasing the levels of 16 amino acid metabolites in flowering leaves (LF) (Table S1). Quercetin-3,4'-O-di-beta-glucopyranoside, taxifolin-3-glucoside, luteolin-7-O-beta-rutinoside, and different amino acids may be metabolites and substrates owing to *DFR* accumulation shortages. Furthermore, *DFR*-KO root (RF) deficit during the blooming stage resulted in a considerable increase in the amount of 11 different amino acids, as well as 4 different flavonoids. *DFR* expression is minimal in wild-type (CK) RF, and we suspect that knocking out *DFR* will have little influence on its metabolites. Similarly, metabolites in mature roots (RM) were less altered in *DFR*-KO. Sugars and organic acids in mature leaves (LM) were reduced in *DFR*-KO, whereas the remainder remained unchanged. Finally, during anthesis, the organs most impacted by *DFR* knockout were leaves (LF) and flowers (FF).

**Fig. 8:**
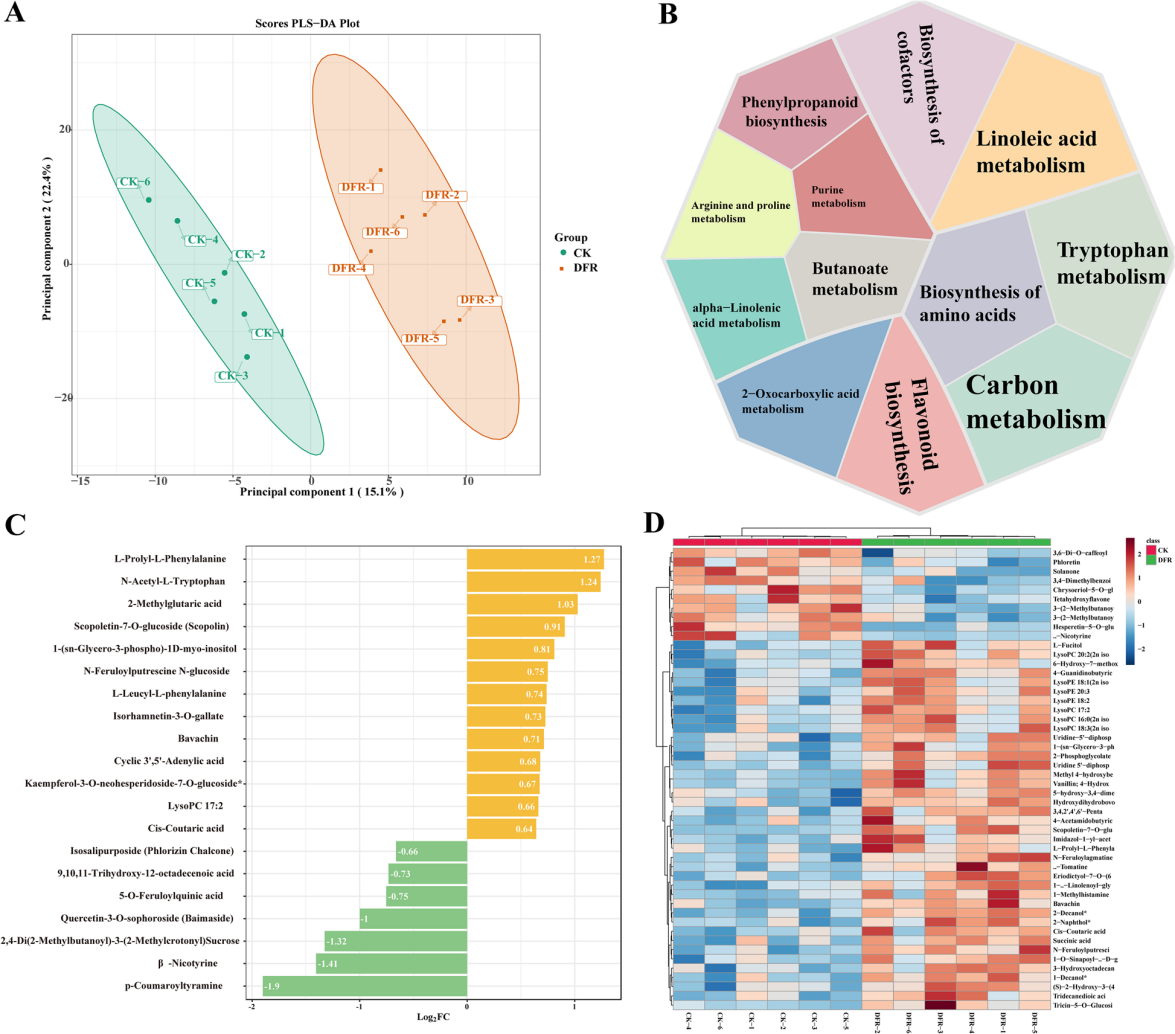
Transcriptome analysis of the LF (leaf samples of flowering stage) stage of CK and *DFR*-KO. **A** PCA of CK and *DFR*-KO data from LF. **B** Differential metabolic pathways in LF. **C** The majority of the 20 metabolites with the greatest changes in LF. **D** Heatmap of the 50 most substantially altered metabolites of LF

Transcriptome analysis revealed that the LF stage and organs were the most severely altered in *DFR*-KO. As a result, we repeated our in-depth LC–MS/MS-based untargeted LF metabolome profiling in CK and *DFR*-KO mice, detecting a total of 1121 metabolites. PCA revealed that the PC1 main component well described the impact of *DFR* deletion on the LF leaf metabolome (Fig. 8A). Meanwhile, functional investigation of differential metabolites between CK and *DFR*-KO revealed that they were mostly involved in amino acid biosynthesis, tryptophan metabolism, and phenylpropanoid production (Fig. 8B). The majority of the 20 metabolites with the greatest changes were flavonoids (Fig. 8C). The analysis of the 50 most substantially altered metabolites revealed that the majority of them (40) were upregulated, while a few were downregulated (10) (Fig. 8D).

### Effect of NtDFR knockout on differential transcription factors in different organs of tobacco development

**Fig. 9:**
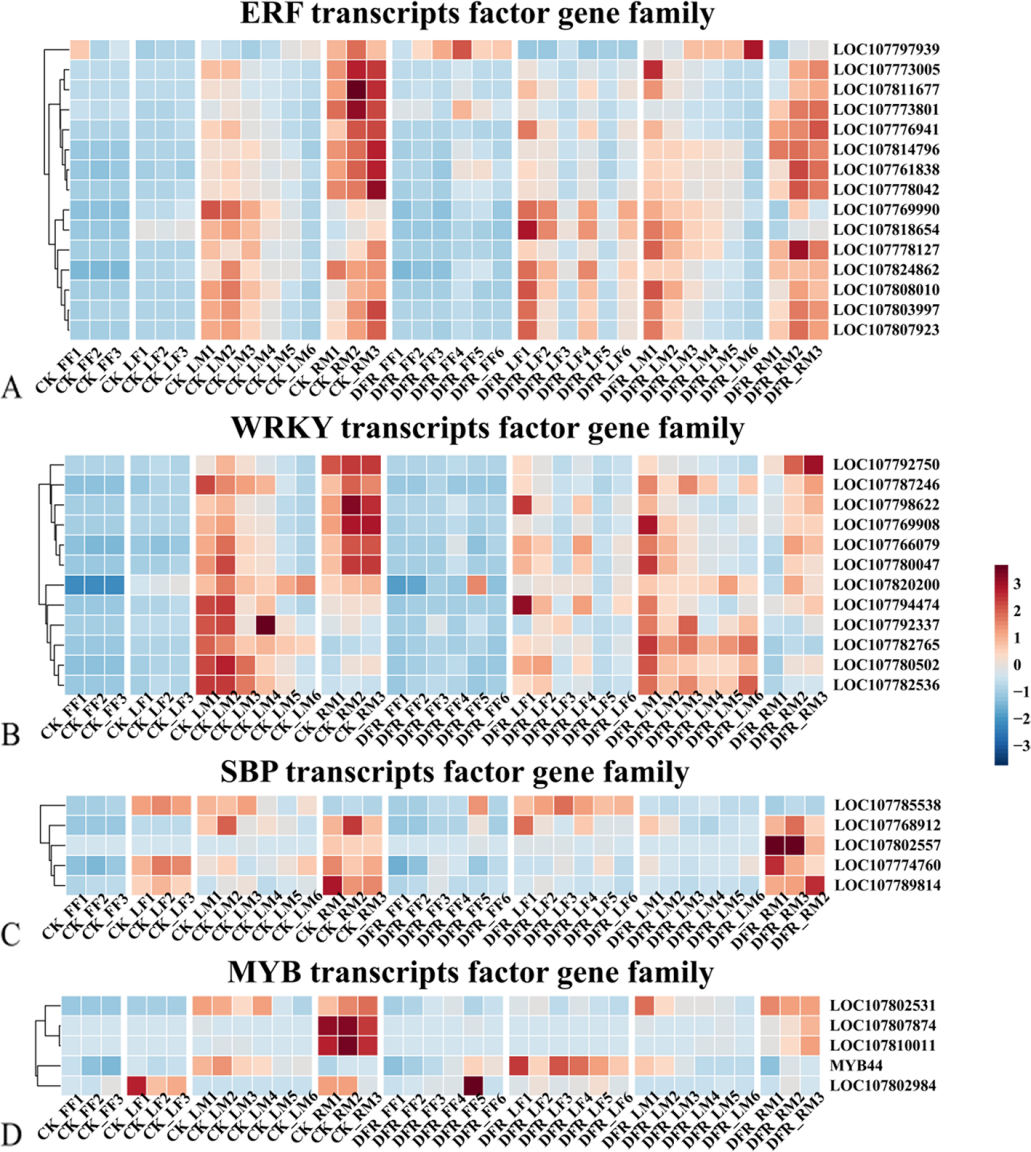
Expression heatmap of the transcription factors *ERF*, *WRKY*, *SBP* and *MYB*

Transcription factors are vital in stress tolerance and growth control in plants. *DFR*-KO altered 37 transcription factors, including 15 *ERF*s, 12 *WRKY*s, 5 *SBP*s, and 5 *MYB*s, according to this research (Fig. 9). The majority of the 37 transcription factors were found to be strongly expressed in mature leaves (LM) and mature roots (RM). In *DFR*-KO, 15 *ERF* transcription factors were upregulated in blooming leaves (LF) and downregulated in mature roots (RM) (Fig. 9A). This might mean that *DFR*-KO plants are more susceptible to ethylene reactions. Meanwhile, 12 *WRKY* transcription factors were upregulated in LF in *DFR*-KO, and the majority of these *WRKY*s were associated with biotic stress resistance (Fig. 9B). The *SBP* transcription factor was mostly downregulated in mature leaves (LM) after *DFR*-KO (Fig. 9C), *MYB* was also downregulated in RM (Fig. 9D).

## Discussion

Anthocyanins are widely distributed in roots, stems, leaves, flowers, fruits and other organs in plants and are secondary metabolites generated in plant morphogenesis or for stress response [26]. It is generally well known that *DFR* genes are the key enzyme genes in the anthocyanin metabolism pathway, and most studies have focused on *DFR* expression in flowers. Transcriptome and metabolome analyses of different parts of *DFR*-KO were performed to explore the effects of *DFR* genes on all sites of tobacco in this study. Knockout mutants of *DFR*s obtained by CRISPR/Cas9, with white flowers that were phenotypically distinct from CK (red flowers). Phenotypic traits (Fig. 2C), the results of Q-PCR (Fig. 2D) and anthocyanin chromatograms (Figure S1) corroborate each other regarding the dependability and validity of the gene editing material used in this investigation. Q-PCR showed that *DFR* genes are expressed to different degrees in different tobacco organs (flower, leaf and root), with significantly reduced expression in the mutant. Despite the trace expression of *DFR*s at the RNA level, the gene function is phenotypically ineffective, presumably because it affects the functional domain of the protein at the transcriptional or protein level.

As an important cash crop, tobacco leaves are an important part of the plant's economic value. We focused on exploring the changes in gene expression and metabolites in tobacco leaf samples during different periods. There were 2898 distinct DEGs in the CK-LF and *DFR*-LF groups, suggesting that the anthesis leaf is the most affected organ following *DFR* knockout. The majority of the upregulated genes in *DFR*-KO were associated with the MAPK signaling pathway, plant–pathogen interaction, carbon fixation in photosynthetic organisms, and the manufacture of ubiquinone and other terpenoid quinones. Carbon fixation in photosynthetic organisms may be closely connected to biomass accumulation in tobacco. Downregulation of oxidative phosphorylation-related genes in *DFR*-KO may indicate an overall decrease in energy metabolism. Photosystem II-related genes were similarly considerably downregulated in *DFR*-KO, suggesting that *DFR*-KO has a significant impact on the photosystem in tobacco leaves, and it may also have a significant impact on the photoreaction stage. Gene functional analysis revealed that flavonoid synthesis, pyridoid synthesis, and the degradation of valine, leucine, and isoleucine were increased in the LM of the *DFR*-KO, suggesting that the massive accumulation of metabolites caused by *DFR* knockout activates other pathways and may further affect the degradation of prerequisite metabolites such as amino acids.

*WRKY* TFs are among the largest families of transcriptional regulators in plants [27]. Many studies suggest transcriptional regulation of *WRKY* TFs in adapting plants to a variety of stressful environments. *WRKY* TFs can regulate diverse biological functions from receptors for pathogen-triggered immunity to modulators of chromatin for specific interactions and signal transfer through a complicated network of genes [28]. In our study, 12 *WRKY* transcription factors were upregulated in LF in *DFR*-KO, and the majority of these *WRKY*s were associated with biotic stress resistance. Studies on *SpWRKY1* showed that it caused an increase in resistance to *Phytophthora infestans* by mediating the regulation of abscisic acid (ABA) biosynthesis genes, and tobacco plants transformed with *SpWRKY1* showed lower contents of malondialdehyde, relative electrolyte leakage and higher antioxidant enzyme peroxidase (*POD*) and superoxide dismutase (*SOD*) and phenylalanine ammonia-lyase (*PAL*) activities, indicating plant resistance to *Phytophthora nicotianae* [28–30]. Meanwhile, the genes related to terpenoid quinones are upregulated, which is also important to combat biological stress. We also found that *DFR*-KOs had better resistance to powdery mildew and black shank in tobacco than the control during the planting process, but this still needs further research.

## Supplementary Information

**Additional file 1:** **Figure S1. **Anthocyanin chromatograms of the flower samples. **Table S1. **Non-targeted metabolome detection using LC-MS/MS of all samples. **Table S2. **Gene number and corresponding names.

## Data Availability

The raw sequence data reported in this paper have been deposited in the Genome Sequence Archive in the National Genomics Data Center, China National Center for Bioinformation/Beijing Institute of Genomics, Chinese Academy of Sciences (GSA: CRA007479), which are publicly accessible at https://ngdc.cncb.ac.cn/gsa/s/04xN4M02.
