# Supplementary figures and images for: Effects of editing DFR genes on flowers, leaves, and roots of tobacco
Source: BMC Plant Biol. 2023 Jul 5;23:349. doi: 10.1186/s12870-023-04307-7 (PMC10320895; doi:10.1186/s12870-023-04307-7)

A

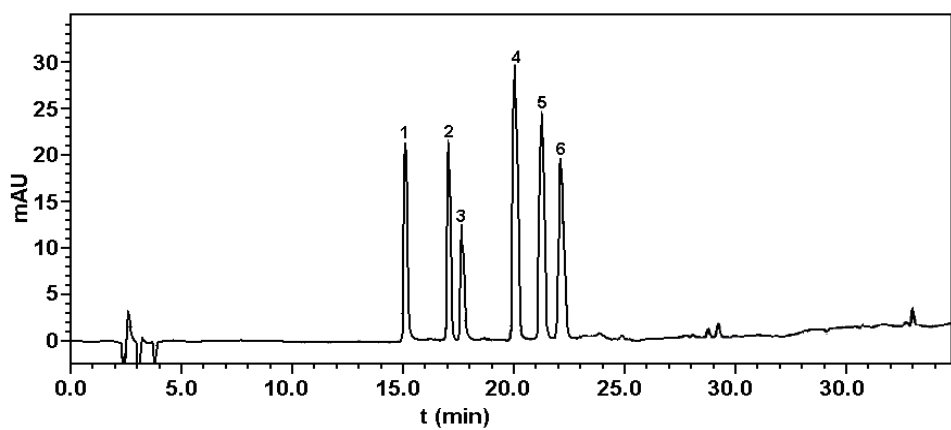

B

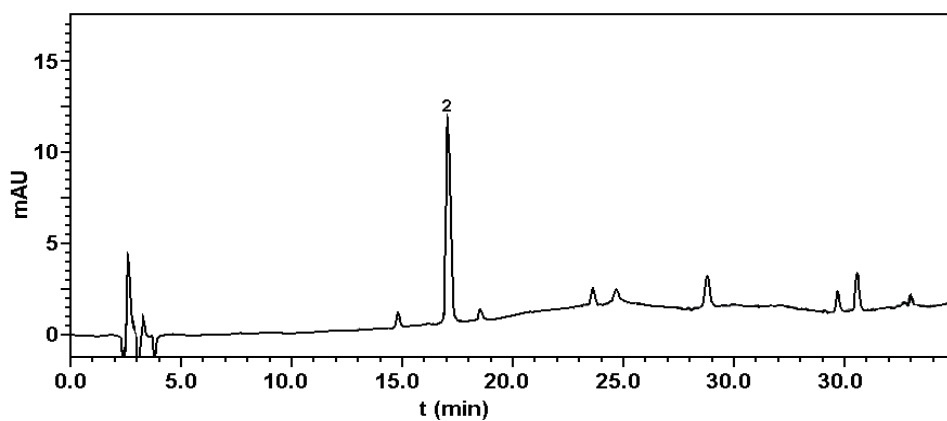

C

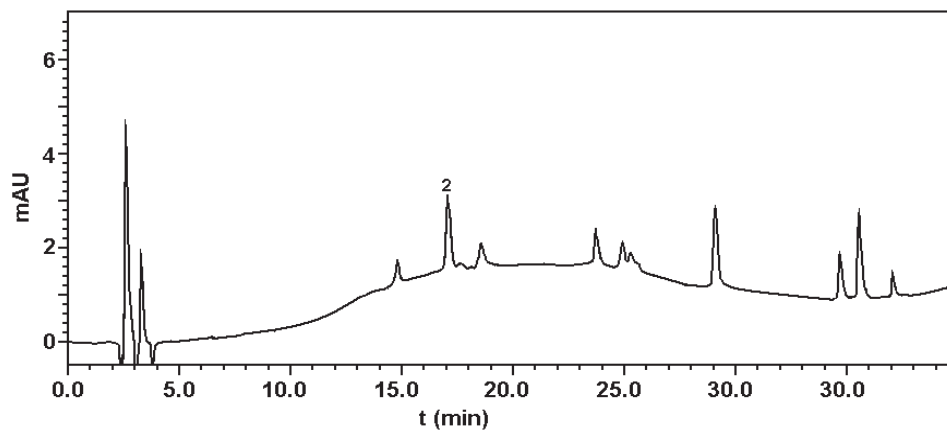

Supplement: Supplementary file 1 — Additional file 1: Figure S1. Anthocyanin chromatograms of the flower samples. Table S1. Non-targeted metabolome detection using LC-MS/MS of all samples. Table S2. Gene number and corresponding names. [file 12870_2023_4307_MOESM1_ESM.zip › figure_S1.pdf]
